# Effects of systemic berberine administration on oral mucosal wound healing: Histopathological and biochemical evaluation in rats

**DOI:** 10.1016/j.jobcr.2026.101457

**Published:** 2026-04-28

**Authors:** Ümit Solmaz, Bilal Ege, Onur Ceylan, Miray Ege, Mahmut Koparal

**Affiliations:** a Department of Oral and Maxillofacial Surgery, Faculty of Dentistry, Adıyaman University, Adıyaman, Türkiye; b Department of Oral and Maxillofacial Surgery, Faculty of Dentistry, Nevsehir Haci Bektas Veli University, Nevsehir, Türkiye; c Department of Medical Pathology, Faculty of Medicine, Atatürk University, Erzurum, Türkiye; d Faculty of Pharmacy, Adıyaman University, Adıyaman, Türkiye

**Keywords:** Berberine, Oral mucosa, Wound healing, Rat model, Histopathology

## Abstract

**Background:**

Berberine is one of the main alkaloids obtained from plants. Numerous experimental and clinical studies have shown that berberine has anti-inflammatory, antioxidant, and immunomodulatory effects. The aim of this study was to investigate the histopathological and biochemical effects of systemic berberine administration on wound healing in the oral mucosa of rats.

**Materials & methods:**

The study was conducted on 42 adult male Wistar Albino rats. The rats were randomly divided into two main groups: control and experimental. In both the experimental and control groups, a mucoperiosteal excisional wound defect with a diameter of 5 mm was created behind the rugae folds in the palatal region. Rats in the experimental group were administered berberine at a rate of 100 mg/kg/day via oral gavage. Control rats received saline. Serum cytokines were analyzed by ELISA. Wound areas dissected from surrounding tissues were histopathologically examined for ulceration, inflammatory cells, necrosis, vascularization, fibroblasts, and edema.

**Results:**

No significant differences between groups in serum cytokine levels. Histopathological examination of polymorphonuclear leukocytes, necrosis, vascularization, and fibroblast levels showed no statistically significant differences between groups on any of the study days. Ulceration was significantly lower in the experimental group on days 3 to 7. Mononuclear cells were significantly lower in the experimental group on day 3, and edema was significantly lower on day 7.

**Conclusion:**

Our study suggests that berberine may have potential positive effects on the early stages of wound healing in the oral mucosa. Further studies are needed to clarify its clinical potential.

## Highlights

• Systemic berberine reduced palatal ulceration during early healing (days 3–7).
• Mononuclear inflammatory cell infiltration was reduced on day 3.
• Histological edema was reduced on day 7 after berberine administration.
• Serum IL-1, IL-2, IL-6, IL-10, TNF-α and NF-κB levels were unchanged between groups.
• Findings support predominantly local tissue effects despite systemic dosing.

## 1 Introduction

Oral mucosa possesses a unique healing ability that develops faster than skin and results in minimal scarring.^1^ This is due to the fact that the oral mucosa and skin exhibit distinct differences at the cellular and molecular levels in terms of wound healing. The oral mucosa is structurally less reactive to inflammation during the wound healing process, resulting in lower macrophage, T cell, and neutrophil infiltration.^2^^,^^3^ Similarly, compared to skin, oral epithelium exhibits lower expression of transforming growth factor beta-1 (TGF-β1), a pro-fibrotic and pro-inflammatory cytokine known to contribute to the formation of hypertrophic scars during wound healing.^4^ In addition, epithelial cell renewal is faster in the oral mucosa (approximately 5–12 days) and has a high regenerative potential; in contrast, renewal occurs more slowly in the skin (approximately 28–40 days) and has a more limited capacity for renewal. In the skin, the inflammatory response is longer-lasting and more intense, and the risk of chronic inflammation is higher. The presence of myofibroblasts is reduced in the oral mucosa, which is associated with low α-smooth muscle actin (α-SMA) expression and minimal fibrosis; in the skin, however, myofibroblasts are more abundant and promote scar formation.

Angiogenesis in the oral mucosa is rapid and transient, primarily regulated by vascular endothelial growth factor (VEGF), and results in early vascular regression. In contrast, angiogenesis in the skin is delayed and characterized by persistent neovascularization. Matrix remodeling in the oral mucosa occurs through a balanced activity of matrix metalloproteinases (MMPs) and tissue inhibitor of metalloproteinases (TIMPs), ensuring effective extracellular matrix (ECM) restoration; in the skin, however, this balance may be disrupted, leading to excessive matrix accumulation.^5^

Despite these advantages, the oral mucosa is frequently damaged during various clinical procedures. Surgical interventions such as palatoplasty, tooth extraction, periodontal surgery, and other dentoalveolar procedures can cause mucosal damage, leading to pain, inflammation, and delayed healing. Additionally, mechanical trauma resulting from chewing, speaking, and oral hygiene practices can further complicate the healing process. Furthermore, the healing of oral wounds occurs in a complex environment characterized by numerous local factors, including high microbial load and infection, ischemia, flap design, and mechanical stress, all of which can negatively impact healing outcomes.^6^

The wound healing process consists of four phases—hemostasis, inflammation, proliferation, and remodeling—which are closely interconnected and occur sequentially. The specific time frames for each of the steps mentioned above are 1–3 days, 3–20 days, 7–40 days, and 40 days to 2 years, respectively.^7^ Following tissue trauma, hemostasis and coagulation mechanisms are rapidly activated to prevent blood loss and protect vital organs by maintaining the integrity of the vascular system.^8^ The hemostatic phase begins within the first few minutes following injury and is characterized by platelet aggregation. During this phase, neutrophil infiltration and the migration of other inflammatory cells to the wound site occur.^9^ Neutrophils are the first immune system cells to arrive at the site of an injury and play a key role in antimicrobial activity through degranulation and phagocytosis.^10^ In addition to their antibacterial effect through phagocytosis and degranulation, neutrophils also release various chemokines to attract monocytes to the site of injury.^11^ After the wound is decontaminated, neutrophils are cleared from the wound site through extrusion, apoptosis, and phagocytosis. In cases of impaired or prolonged wound healing, neutrophils persist in the wound site for an extended period and create a chronic wound environment through continuous protease production.^12^^,^^13^

Approximately 48 to 72 h after an injury, monocytes migrate to the wound site and primarily differentiate into M1-type macrophages—described as “pro-inflammatory”—serving as the predominant cell type during the inflammatory phase of wound healing.^14^ M1 cells possess remarkable phagocytic properties, phagocytosing neutrophils that have undergone apoptosis and eliminating any pathogens or debris present in the wound.^15^ After fulfilling these roles, M1 macrophages are converted into M2-type macrophages, which are described as “anti-inflammatory” and play a role in initiating fibroblast proliferation and angiogenic processes, synthesizing anti-inflammatory mediators, and producing the ECM.^16^

The proliferation phase involves the migration and proliferation of fibroblasts, keratinocytes, and endothelial cells to the wound site, which are necessary for re-epithelialization and the formation of granulation tissue.^17^ Fibroblasts are the cells responsible for producing new ECM. Collagen is the primary component of mature connective tissue scar tissue, which is secreted by fibroblasts. Additionally, fibroblasts synthesize type III collagen, which constitutes the predominant form of granulation tissue.^18^

In the early stages of wound healing, while cells causing inflammation are active within the fibrin-rich temporary matrix, fibroblasts become activated starting around days 3–5 due to the induction of growth factors such as PDGF and TGF-β secreted by macrophages present in the wound, and they proliferate by infiltrating the wound site from the surrounding healthy tissue. Consequently, during this phase of wound healing, fibroblasts become the primary cell group playing an active role in the wound site.^19^

Reduced blood flow in the wound site leads to hypoxia, which triggers an angiogenic response. Cells in the hypoxic wound bed secrete various proangiogenic factors, such as hypoxia-inducible factor-1α (HIF-1α) and VEGF, to stimulate endothelial cell proliferation and migration. This, in turn, facilitates the production of a large amount of new vascular structures with high vascular permeability, which supply nutrients and immune cells to the granulation tissue.^20^ Angiogenesis is a key component of the proliferative phase and ensures the delivery of sufficient oxygen and nutrients to cells in the wound tissue to support tissue regeneration.^21^^,^^22^

In the final stage of wound healing, the repaired tissue undergoes a phase of remodeling and maturation that begins approximately three weeks after the injury and can last up to two years.^23^ Protease activity, particularly that of MMPs, helps facilitate healing during the remodeling phase by maintaining a balance between the accumulation and degradation of the ECM.^24^ However, in cases where healing takes a long time, pro-inflammatory cytokines can trigger high levels of MMP production, leading to excessive degradation of the extracellular matrix.^25^ During this phase of wound healing, the wound becomes less vascularized and the fibrillar network of the ECM becomes more organized.^23^ Wound healing's remodeling phase refers to the transition from transient granulation tissue to a structurally and functionally mature ECM and is characterized by the restoration of tissue integrity and mechanical strength.^9^

The wound healing process, involving tissue repair, is a continuous and dynamic event controlled by numerous growth factors and other cytokines such as TNF-α and interleukins.^26^ Depending on their roles, cytokines can also be classified as pro-inflammatory or anti-inflammatory.^27^ Pro-inflammatory cytokines, including TNF-α, IL-1β, IL-6, IL-8, IL-12 and interferons, cause inflammatory reactions and tend to activate immune system cells. In contrast, anti-inflammatory cytokines such as IL-1 receptor antagonist (IL-1 RA), IL-4, IL-6, IL-10, IL-11, IL-13, and TGF-β inhibit inflammation and suppress immune cells.^28^ When the balance between pro-inflammatory and anti-inflammatory cytokines is disrupted, wound healing is negatively affected, and the inflammatory phase is prolonged, potentially leading to chronic wound healing.^29^ Therefore, effectively controlling inflammation is critical for the smooth healing of soft tissue wounds following oral surgical procedures.

The successful repair or regeneration of defects following oral surgery is crucial for both the patient and the surgeon. To this end, various methods such as growth factors, low-dose laser, hyperbaric oxygen therapy, and ozone therapy have been applied to ensure uncomplicated wound healing 30, 31, 32, 33. However, these methods have significant disadvantages such as high cost, limited accessibility, and technical complexity. Therefore, in recent years, there has been a growing interest in phytotherapeutic agents as potential complementary or alternative strategies in wound healing 34, 35, 36, 37. Throughout history, plants have been widely used in wound treatment due to their anti-inflammatory, antioxidant, and analgesic effects.^38^ However, comprehensive preclinical and clinical studies are necessary to scientifically demonstrate the effectiveness of herbal medicines. These studies should not only encourage patients and healthcare professionals to use herbal medicines, but also provide sufficient and reliable information about the correct methods of use, possible side effects, and potential risks of these agents.^39^^,^^40^

In this context, one of the herbal compounds whose therapeutic effects have been most extensively researched is berberine (BBR). Berberine, Annonaceae (Annickia, Coelocline, Rollinia, and Xylopia), Berberidaceae (Berberis, Caulophyllum, Jeffersonia, Mahonia, Nandina, and Sinopodophyllum), Menispermaceae (Tinospora), Papaveraceae (Argemone, Bocconia, Chelidonium, Corydalis, Eschscholzia, Glaucium, Hunnemannia, Macleaya, Papaver, and Sanguinaria), Ranunculaceae (Coptis, Hydrastis, and Xanthorhiza), and Rutaceae (Evodia, Phellodendron, and Zanthoxyllum). The genus Berberis, found in the Berberidaceae family, is the most widely known natural source of BBR. Plants containing berberine have traditionally been used by people in various regions of the world to treat inflammatory conditions, skin diseases, mouth ulcers, to promote wound healing, reduce fever, relieve pain, treat eye conditions, treat tumors, and address digestive and respiratory tract diseases, as well as microbial infections.^41^ Berberine, *Hydrastis canadensis* (goldenseal), *Coptis chinensis* (Coptis or golden thread), *Berberis aquifolium* (Oregon grape), *Berberis vulgaris* (barberry), and *Berberis aristata* (turmeric tree), an Indian species, among others; it is an active compound found in the roots, rhizomes, and bark of many medically important plants.^42^

These plants have been used in traditional medicine for many years, particularly in Asia (especially China and India), the Middle East, Europe, and certain regions of South America. Due to their antimicrobial, anti-inflammatory, and antiparasitic effects, they have a wide range of applications. Their broad geographic distribution and long-standing use in various traditional medical systems contribute to their easy availability in many regions.^41^

Although berberine has a long history of use in traditional medicine, systematic research on its translational potential has increased significantly in recent years. Berberine is a quaternary isoquinoline alkaloid isolated from several medicinal plants, including *Hydrastis canadensis*, *Berberis aristata*, *Coptis chinensis*, Coptis rhizome, *Coptis japonica*, *Phellodendron amurense*, and *Phellodendron chinense* schneid.^43^ Recent studies have shown that BBR possesses numerous pharmacological activities, including antimicrobial, anti-inflammatory, antioxidant, hypoglycemic, hypolipidemic, and hepatoprotective properties.^42^^,^^44^ BBR, particularly due to its anti-inflammatory,^45^ antioxidant,^46^ and antimicrobial^47^ effects, suggests that it may suppress excessive immune responses during the early inflammatory phase of wound healing; thus, it could limit tissue damage and facilitate the repair process.

Despite the abundance of evidence on this subject, studies investigating the effects of BBR on wound healing have generally focused on skin or gastrointestinal system models and have mostly tested local application routes. Regarding experimental wound models of the oral mucosa, studies evaluating the effects of systemic BBR application on both the early and late phases of wound healing are quite rare. Moreover, the influence of BBR's pharmacokinetic limitations, including low bioavailability and rapid elimination, on histopathological and biochemical outcomes has not been sufficiently elucidated.

The aim of this study was to investigate the effects of BBR on wound healing using histological and biochemical analyses in a rat model of a mucoperiosteal soft tissue defect created in the palatal mucosa and allowed to heal by secondary intention.

## 2 Materials and methods

### 2.1 Sample size calculation and ethical approval

An a priori sample size estimation was performed using G∗Power (version 3.1) for an independent two-group comparison. Assuming a large effect size (Cohen's d = 0.8), a two-sided α level of 0.05, and 80% power, a total of 42 animals (21 per group) was planned. Because animals were sacrificed at predefined time points (days 3, 7, and 14), outcomes were analyzed separately at each time point (n = 7 per group), and the study was therefore designed and interpreted as exploratory with respect to time point–specific comparisons.

The study was approved by the Adıyaman University Local Ethics Committee for Animal Experiments (Protocol No: 2021/037; Meeting Date: 30 September 2021). A total of 42 adult male Wistar Albino rats, aged 4–6 months, with a mean body weight of 350–400 g, were included in the study. All animals were experimentally naive and had not been used in any previous research. Animals were acclimatised for at least 7 days before experiments and housed at 22–24 °C, 40–60% humidity, 12-h light/dark cycle, with environmental enrichment.

Following ethical approval, the study was submitted to the Scientific Research Projects Unit of Adıyaman University (ADYU-BAP) and was accepted under project number DHFDUP/2021-0005. The study was financially supported by ADYU-BAP.

### 2.2 Study design and allocation of study groups

The rats included in the study were randomly allocated into two main groups: a control group and an experimental group. Randomisation was performed using a computer-generated random sequence. Each main group was further divided into three subgroups according to the sacrifice time points (3rd, 7th, and 14th days), with seven rats assigned to each subgroup (Table 1).

**Table 1:** Schematic representation of the experimental study groups.

Study groups	Sacrifice time points		
	Day 3	Day 7	Day 14
**Control group**	n = 7	n = 7	n = 7
**Experimental group**	n = 7	n = 7	n = 7

No animals were excluded from the analysis other than two control rats that died due to anaesthesia-related complications on day 14. If incidental losses occurred, analyses were conducted with the available sample size for that time point.

### 2.3 Surgical procedure

All surgical procedures were performed under the supervision of a veterinarian. Prior to wound creation, the rats were placed under general anaesthesia by intramuscular injection of ketamine hydrochloride (30 mg/kg; Ketalar®, Eczacıbaşı) and xylazine hydrochloride (5 mg/kg; Rompun®, Bayer). All surgical interventions were carried out under aseptic conditions.

**Fig. 1:**
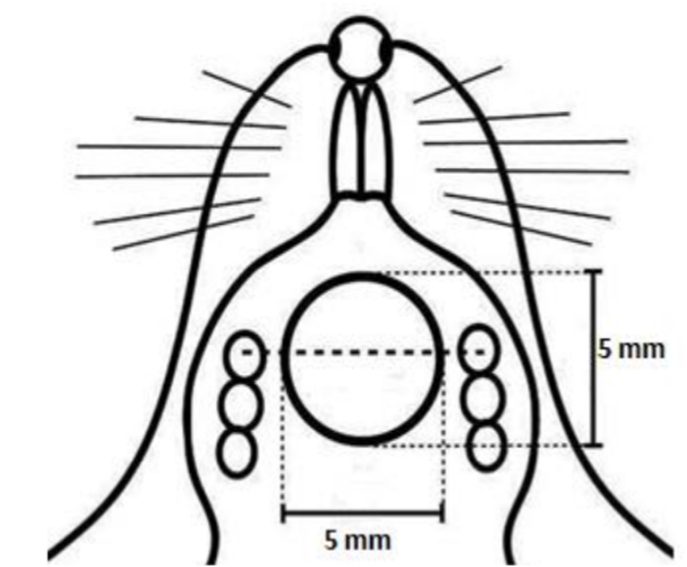
Experimental palatal mucoperiosteal wound model.

In the maxillary palatal region, a full-thickness mucoperiosteal wound with a diameter of 5 mm was created using a biopsy punch posterior to the palatal rugae, as previously described in the literature (Fig. 1).^48^

Following haemostasis, the wound area was left to heal by secondary intention. The day on which the wound was created was designated as day 0. Following surgery, all animals were monitored until full recovery from anaesthesia and were then returned to their cages. Throughout the postoperative period, the rats were observed daily for general health status, signs of distress, infection, or complications related to the surgical procedure. Animals were provided with standard laboratory chow and water *ad libitum* under controlled environmental conditions. No postoperative complications requiring exclusion from the study were observed.

Postoperative analgesia was provided using paracetamol (Parol®, Atabay, Turkey), administered via the drinking water for the first 72 h postoperatively. Based on average daily water consumption in adult rats, the estimated paracetamol intake corresponded to approximately 200 mg/kg/day, consistent with doses commonly reported for analgesia in rat models. Analgesic-containing water was provided *ad libitum*, and animals were monitored daily for signs of pain or distress.

### 2.4 Drug administration

From the day of wound creation, berberine (100 mg/kg; Kenay GmbH, Berlin, Germany) solution was administered to the rats in the experimental groups once daily by oral gavage at the same time each day. Rats in the control groups received a placebo sterile saline solution via the same administration route.

### 2.5 Collection of blood and tissue samples

Euthanasia was performed on days 3, 7, and 14 under deep general anaesthesia induced by intramuscular injection of ketamine hydrochloride (30 mg/kg; Ketalar®, Eczacıbaşı) and xylazine hydrochloride (5 mg/kg; Rompun®, Bayer). Following confirmation of a surgical plane of anaesthesia, blood samples were collected by cardiac puncture as part of terminal exsanguination. Subsequently, in accordance with institutional and international animal welfare guidelines, the rats were humanely euthanized by cervical dislocation, a physical method. Serum samples were obtained by centrifuging the collected blood at 3000×*g* for 10 min and stored at −80 °C until the day of biochemical analysis.

On the days of sacrifice, full-thickness tissue samples measuring 1 × 1 cm, including the surrounding healthy tissue, were taken from the wounded palate tissues for histopathological examination.

### 2.6 Evaluation methods

Due to the exploratory nature of the study and the rarity of research on this topic, a primary outcome variable was not identified. The potential effects of berberine on multiple histopathological and biochemical parameters were investigated.

### 2.7 Histopathological evaluation

Tissue samples obtained from the palatal wound area of the control and experimental groups on days 3, 7, and 14 were subjected to routine tissue processing procedures. The prepared slides were stained with Hematoxylin-Eosin (H&E) to monitor inflammatory and proliferative changes in wound healing and evaluated under a light microscope. All samples were photographed after being magnified 100 times under the microscope.

Histolopathogical examination was performed by the same pathologist without knowing which sample belonged to which study group. In the histopathological examination, polymorphonuclear leukocytes (PMNL), mononuclear cells (MNCs), ulceration, necrosis, vascularization, fibroblast count, and edema level were assessed.

As is commonly done in experimental wound healing studies, a semi-quantitative scoring system (0–3 scale) was used for all parameters examined; here, 0 = absent, 1 = mild, 2 = moderate, and 3 = severe. Scoring was based on the relative density, distribution, and morphological appearance of the relevant histopathological findings in the examined field. Mild changes were defined as sparse, scattered findings accompanied by minimal tissue involvement; moderate changes were defined as clearly identifiable findings with a more widespread distribution; and severe changes were defined as dense, widespread, and scattered findings, typically accompanied by marked tissue changes. Inflammatory cell infiltration (PMNL and MNCs) was assessed based on the number of inflammatory cells and their distribution pattern. Fibroblast proliferation was assessed based on the abundance of fibroblasts and extracellular matrix formation, while vascularization was assessed based on the presence and prominence of newly formed blood vessels. Edema was assessed based on the degree of interstitial fluid accumulation and tissue separation. Ulceration and necrosis were assessed based on the degree of epithelial loss and structural tissue damage. This semi-quantitative histopathological approach is consistent with previously reported experimental wound healing studies.^49^

In addition to the semi-quantitative assessment, quantitative histomorphometric analysis was performed using ImageJ software (National Institutes of Health, USA). As described in previous experimental wound models, ImageJ was used for area-based measurements to support the evaluation of tissue morphology, particularly vascularization and fibroblast density.^50^^,^^51^

### 2.8 Biochemical analysis

Serum samples were thawed from −80 °C to room temperature for analysis. Serum levels of tumor necrosis factor alpha (TNF-α), nuclear factor kappa B (NF-κB), interleukin IL-1, IL-2, IL-6, and IL-10 were determined using rat-specific ELISA kits (Elabscience Biotechnology Inc., Houston, TX, USA) according to the manufacturer's instructions. Absorbance values were measured using a BioTek ELx800 microplate reader (BioTek Instruments, Winooski, VT, USA), and cytokine concentrations were calculated from standard curves generated for each assay.

### 2.9 Statistical analysis

Statistical analyses were performed using IBM SPSS Statistics for Windows, version 28.0 (IBM Corp., Armonk, NY, USA). Data distribution was assessed using the Shapiro–Wilk test. Histopathological outcomes, which were obtained using an ordinal semi-quantitative scoring system, were compared between the experimental and control groups at each sacrifice time point using the Mann–Whitney *U* test. For biochemical (ELISA) outcomes, between-group comparisons at each time point were conducted using an independent-samples *t*-test for normally distributed variables and the Mann–Whitney *U* test for non-normally distributed variables. Where multiple comparisons were performed across biochemical outcomes, p values were adjusted using the Benjamini–Hochberg false discovery rate (FDR) procedure, and FDR-adjusted p values were considered for statistical inference. All statistical tests were two-sided, and a p value of <0.05 was considered statistically significant. No repeated-measures analyses were performed, as different animals were sacrificed at each predefined time point.

## 3 Results

### 3.1 Observational findings related to the rats

Throughout the experimental period, the general health status, food and water intake, body weight, and palatal condition of all rats were monitored daily. On day 14, two control rats died due to anaesthesia-related complications; no other exclusions occurred. Accordingly, the day-14 control subgroup comprised five animals for outcome analyses.

### 3.2 Histopathological findings

To evaluate wound healing, histopathological assessments were performed to compare the control and experimental groups at different time points with respect to ulceration, polymorphonuclear leukocytes (PMNL), mononuclear cells (MNCs), necrosis, vascularization, fibroblast count, and edema. According to the statistical analysis, significant differences were observed between the two groups on day 3 for the parameters of ulceration and MNCs (p < 0.05) (Table 2).

**Table 2:** Comparison of histopathological parameters between control and experimental groups on day 3.

Parameter	Group	None n (%)	Mild n (%)	Moderate n (%)	Severe n (%)	χ^2^	p value
Ulceration	Control	1 (14.3)	0 (0)	2 (28.6)	4 (57.1)	8.667	0.034∗
	Experimental	0 (0)	3 (42.9)	4 (57.1)	0 (0)		
PMNL infiltration	Control	0 (0)	1 (14.3)	2 (28.6)	4 (57.1)	5.667	0.058
	Experimental	0 (0)	3 (42.9)	4 (57.1)	0 (0)		
Mononuclear cell infiltration	Control	1 (14.3)	6 (85.7)	0 (0)	0 (0)	10.57	0.005∗
	Experimental	0 (0)	6 (85.7)	1 (14.3)	0 (0)		
Necrosis	Control	0 (0)	1 (14.3)	2 (28.6)	4 (57.1)	5.667	0.058
	Experimental	0 (0)	3 (42.9)	4 (57.1)	0 (0)		
Vascularization	Control	0 (0)	1 (14.3)	2 (28.6)	4 (57.1)	5.667	0.058
	Experimental	0 (0)	3 (42.9)	4 (57.1)	0 (0)		
Fibroblast count	Control	2 (28.6)	5 (71.4)	0 (0)	—	2.33	0.126
	Experimental	0 (0)	7 (100)	0 (0)	—		
Edema	Control	2 (28.6)	4 (57.1)	1 (14.3)	—	0.444	0.800
	Experimental	1 (14.3)	5 (71.4)	1 (14.3)	—		

**Statistical analysis:** Differences between groups were analyzed using the chi-square test. Data are presented as number (percentage). Sample size was n = 7 per group at days 3 and 7, and n = 5 (control) and n = 7 (experimental) at day 14 due to mortality. PMNL, polymorphonuclear leukocytes. ∗Statistically significant at p < 0.05.

Fig. 2A shows the severe PMNL levels observed in rats in the control group; Fig. 2B shows the mild PMNL levels observed in rats in the experimental group. Fig. 3A shows the severe MNC levels observed in rats in the control group; Fig. 3B shows the mild MNC levels observed in rats in the experimental group.

**Fig. 2A:**
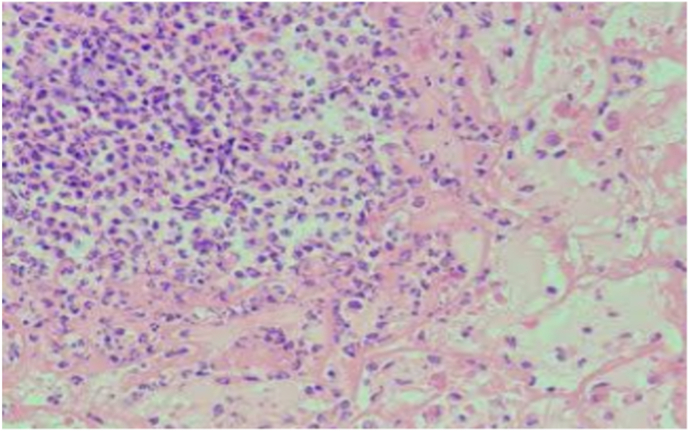
Severe PMNL levels in the control group on day 3 (H&E, x100).

**Fig. 2B:**
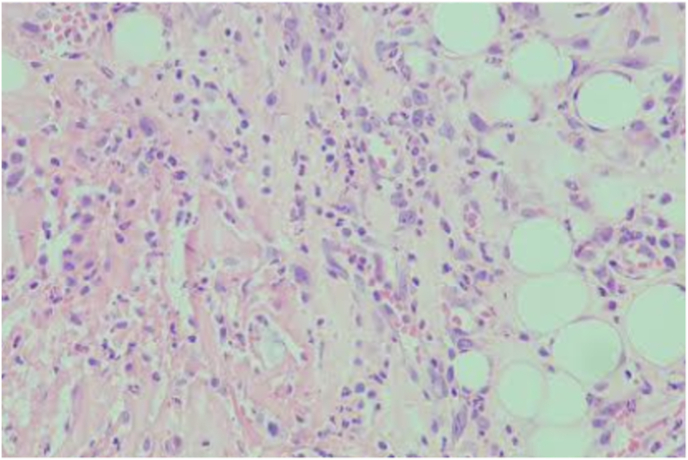
Mild PMNL levels in the experimental group on day 3 (H&E, x100).

**Fig. 3A:**
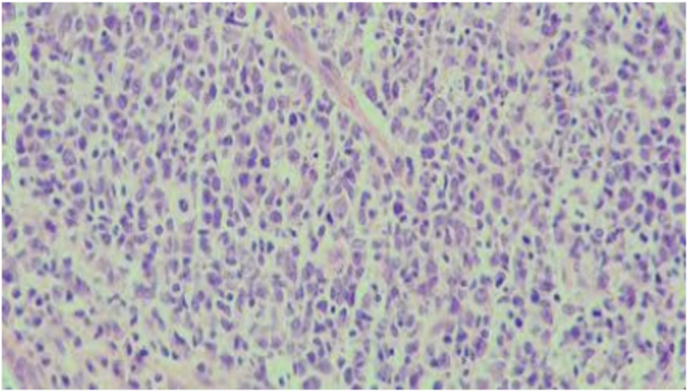
Severe MNC levels in the control group on day 3 (H&E, x100).

**Fig. 3B:**
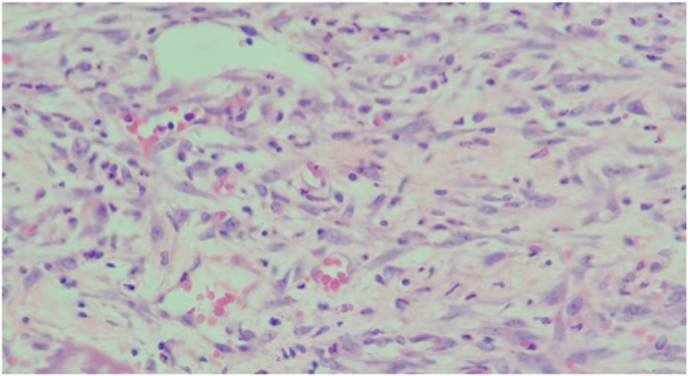
Mild MNC levels in the experimental group on day 3 **(**H&E, x100).

On day 7, ulceration and edema scores in the experimental group were found to be significantly lower compared to the control group (p < 0.05) (Table 3).

**Table 3:** Comparison of histopathological parameters between control and experimental groups on day 7.

Parameter	Group	None n (%)	Mild n (%)	Moderate n (%)	Severe n (%)	χ^2^	p value
Ulceration	Control	0 (0)	6 (85.7)	1 (14.3)	0 (0)	6.00	0.049∗
	Experimental	4 (57.1)	3 (42.9)	0 (0)	0 (0)		
PMNL infiltration	Control	0 (0)	4 (57.1)	3 (42.9)	0 (0)	4.40	0.110
	Experimental	1 (14.3)	6 (85.7)	0 (0)	0 (0)		
Mononuclear cell infiltration	Control	0 (0)	7 (100)	0 (0)	0 (0)	1.07	0.299
	Experimental	1 (14.3)	6 (85.7)	0 (0)	0 (0)		
Necrosis	Control	0 (0)	6 (85.7)	1 (14.3)	0 (0)	4.400	0.111
	Experimental	3 (42.9)	4 (57.1)	0 (0)	0 (0)		
Vascularization	Control	0 (0)	6 (85.7)	1 (14.3)	0 (0)	0.024	0.987
	Experimental	1 (14.3)	5 (71.4)	1 (14.3)	0 (0)		
Fibroblast count	Control	0 (0)	3 (42.9)	4 (57.1)	—	1.810	0.404
	Experimental	1 (14.3)	4 (57.1)	2 (28.6)	—		
Edema	Control	0 (0)	7 (100)	0 (0)	—	7.778	0.005∗
	Experimental	5 (71.4)	2 (28.6)	0 (0)	—		

**Statistical analysis:** Differences between groups were analyzed using the chi-square test. Data are presented as number (percentage). Sample size was n = 7 per group at days 3 and 7, and n = 5 (control) and n = 7 (experimental) at day 14 due to mortality. PMNL, polymorphonuclear leukocytes. Statistically significant at p < 0.05.

Fig. 4A shows the ulceration in the control group, and Fig. 4B shows the ulceration in the experimental group. Fig. 5A shows the edema in the control group, and Fig. 5B shows the edema in the experimental group.

**Fig. 4A:**
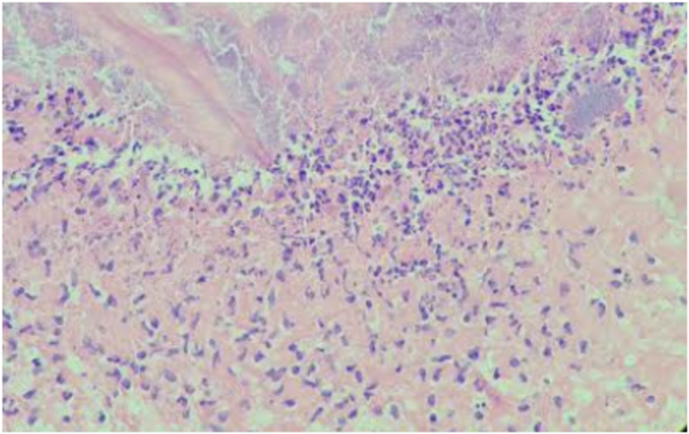
Severe ulceration in the control group on day 3 (H&E x100).

**Fig. 4B:**
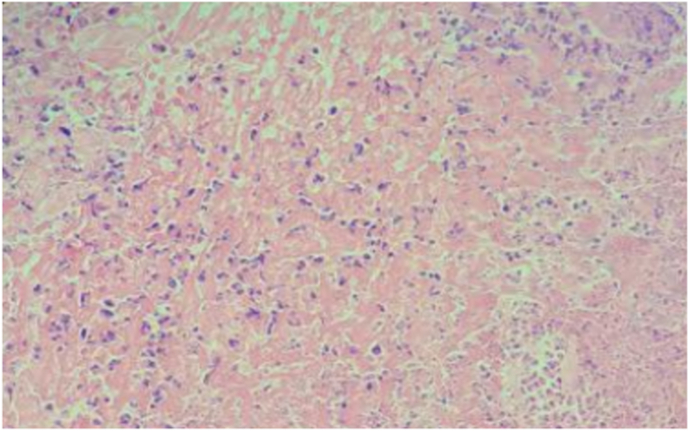
Mild ulceration in the experimental group on day 3 (H&E x100).

**Fig. 5A:**
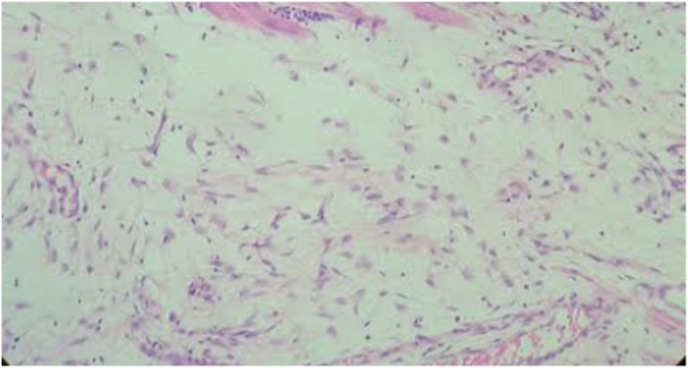
Edema in the control group on day 7 (H&E x100).

**Fig. 5B:**
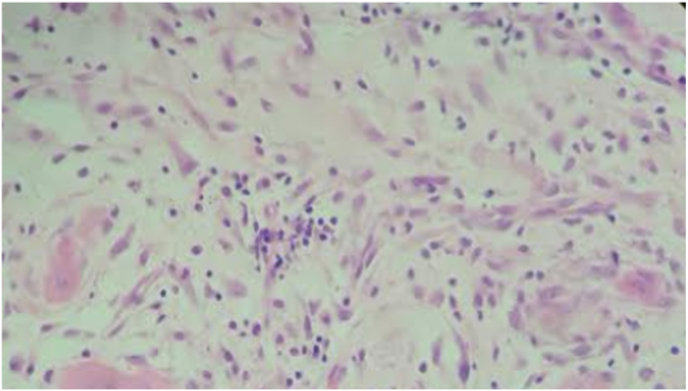
Edema in the control group on day 7 (H&E x100).

In contrast, no statistically significant differences were observed between the control and experimental groups for any of the evaluated parameters on day 14 (Table 4).

**Table 4:** Comparison of histopathological parameters between control and experimental groups on day 14.

Parameter	Group	None n (%)	Mild n (%)	Moderate n (%)	Severe n (%)	χ^2^	p value
Ulceration	Control	1 (20.0)	0 (0)	4 (80.0)	0 (0)	3.771	0.152
	Experimental	2 (28.6)	3 (42.9)	2 (28.6)	0 (0)		
PMNL infiltration	Control	0 (0)	0 (0)	5 (100)	0 (0)	2.857	0.239
	Experimental	2 (28.6)	1 (14.3)	4 (57.1)	0 (0)		
Mononuclear cell infiltration	Control	0 (0)	1 (20.0)	3 (60.0)	1 (20.0)	3.977	0.264
	Experimental	2 (28.6)	3 (42.9)	2 (28.6)	0 (0)		
Necrosis	Control	0 (0)	1 (20.0)	4 (80.0)	0 (0)	2.890	0.235
	Experimental	3 (42.9)	1 (14.3)	3 (42.9)	0 (0)		
Vascularization	Control	0 (0)	2 (40.0)	3 (60.0)	0 (0)	1.714	0.424
	Experimental	2 (28.6)	2 (28.6)	3 (42.9)	0 (0)		
Fibroblast count	Control	0 (0)	2 (40.0)	3 (60.0)	—	0.171	0.678
	Experimental	0 (0)	2 (28.6)	5 (71.4)	—		
Edema	Control	1 (20.0)	4 (80.0)	0 (0)	—	3.086	0.079
	Experimental	5 (71.4)	2 (28.6)	0 (0)	—		

**Statistical analysis:** Differences between groups were analyzed using the chi-square test. Data are presented as number (percentage). Sample size was n = 7 per group at days 3 and 7, and n = 5 (control) and n = 7 (experimental) at day 14 due to mortality. PMNL, polymorphonuclear leukocytes. Statistically significant at p < 0.05.

**Fig. 6:**
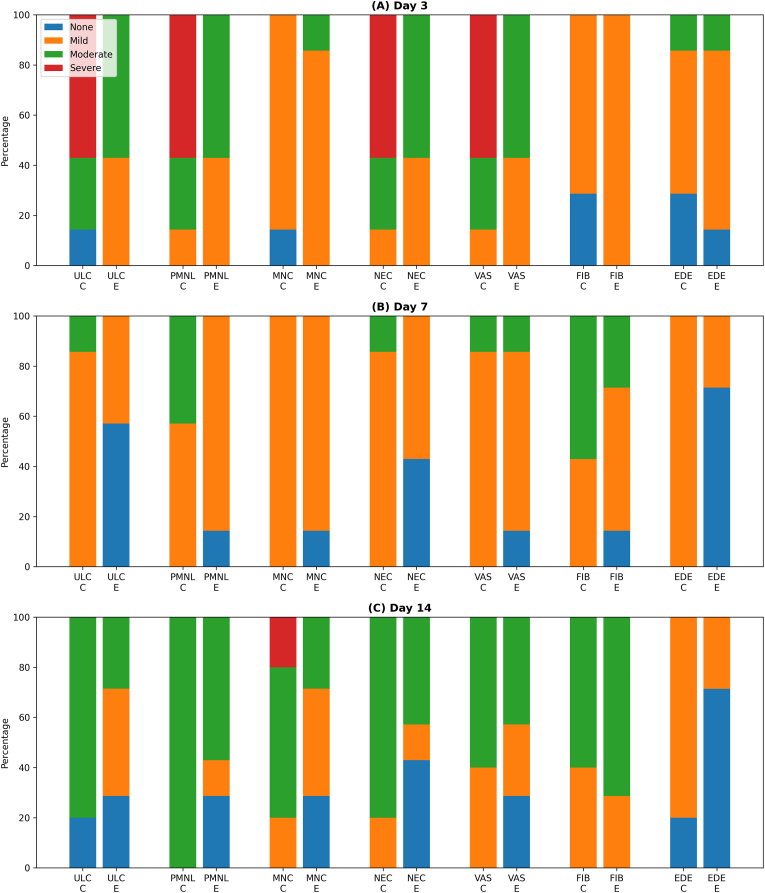
Distribution of histopathological scores (none, mild, moderate, and severe) at different time points in the control (C) and experimental (E) groups. (A) Day 3, (B) Day 7, and (C) Day 14. The evaluated parameters include ulceration (ULC), polymorphonuclear leukocyte infiltration (PMNL), mononuclear cell infiltration (MNC), necrosis (NEC), vascularization (VAS), fibroblast count (FIB), and edema (EDE). Data are presented as percentages.

In summary, statistically significant differences were observed between the groups regarding ulceration and MNC on day 3, and regarding ulceration and edema on day 7 (p < 0.05). The distribution of histopathological scores across groups and time points is presented graphically in Fig. 6.

### 3.3 Biochemical findings

Serum levels of interleukin (IL)-1, IL-2, IL-6, IL-10, tumour necrosis factor-alpha (TNF-α), and nuclear factor kappa B (NF-κB), measured by ELISA in serum samples obtained on the sacrifice days, did not show statistically significant differences between the experimental and control groups.

## 4 Discussion

Phytotherapy is a treatment approach based on the use of plant-derived agents for various disease conditions and has long been incorporated into both clinical and experimental studies.^52^ Owing to their relatively easy accessibility from natural sources and cost-effectiveness, interest in herbal agents has steadily increased.^53^ In particular, the antioxidant and anti-inflammatory properties of phytotherapeutic agents have been shown to limit tissue damage and support wound healing by neutralising reactive oxygen species released during phagocytosis by neutrophils and macrophages in the inflammatory phase of wound healing.^54^ These characteristics underscore the potential therapeutic role of phytotherapeutic agents in wound healing processes.

In this context, berberine (BBR), an isoquinoline alkaloid, has gained increasing attention in wound healing and inflammation models due to its anti-inflammatory, antioxidant, antimicrobial, and immunomodulatory properties.

Peng et al.^55^ demonstrated that BBR injection suppressed NF-κB protein expression in ovarian tissue and significantly inhibited IL-6 and IL-1β mRNA expression in rats with induced premature ovarian failure. Similarly, Chen et al.^45^ reported that BBR suppressed TNF-α, IL-6, and MCP-1 expression in macrophages in a dose-dependent manner in a cell culture model. These studies reveal that the anti-inflammatory effects of BBR are particularly pronounced at the tissue and cellular levels.

In the present study, no statistically significant difference was found between the experimental and control groups in terms of IL-1, IL-2, IL-6, IL-10, TNF-α and NF-κB levels measured by ELISA method from serum samples obtained on sacrifice days. This suggests that the anti-inflammatory effects of BBR may occur at the local tissue level rather than the systemic circulation. Indeed, in a randomized, double-blind phase I clinical trial conducted in patients with ulcerative colitis, it was reported that BBR treatment applied for three months significantly reduced tissue-level inflammation, while it did not cause a significant change in the tissue expression of NF-κB and COX-2 and plasma cytokine levels.^56^ These findings support the importance of the methodological difference between serum and tissue-level assessments.

Furthermore, Tan et al.^57^ demonstrated that following oral administration of BBR in Sprague-Dawley rats, the concentrations of BBR and its bioactive metabolites in organ tissues were higher than in blood. This pharmacokinetic feature can be considered an important factor that may explain why no significant change in serum cytokine levels was detected in our study. Therefore, the findings suggest that the anti-inflammatory effects of BBR occur mainly through local tissue accumulation and cellular signaling pathways, and that the parameters measured in systemic circulation may not adequately reflect this effect.

Previous studies in the literature have reported that BBR application in damaged tissues significantly reduces neutrophil infiltration and thus suppresses the inflammatory response.^58^^,^^59^ In our study, PMNL and MNC counts were analyzed to evaluate the anti-inflammatory effect of BBR. According to the data obtained, a decrease close to the limit of statistical significance was detected in the experimental group in terms of PMNL count on day 3 compared to the control group (p = 0.058). In contrast, there was no statistically significant difference between the groups in terms of PMNL counts on days 7 and 14.

When the number of MNCs was evaluated, a statistically significant decrease was observed in the experimental group compared to the control group on day 3 (p = 0.0051). However, on days 7 and 14, when the inflammatory response naturally decreased, no significant difference was observed between the groups in terms of MNC numbers.

Taken together, these findings suggest that BBR may limit excessive inflammatory cell infiltration during the early inflammatory phase of wound healing, particularly within the first three days, thereby supporting early wound repair. One of the key strengths of this study is the evaluation of berberine's effects during the early inflammatory phase of wound healing in the oral mucosa. The early phase of wound healing plays a critical role in determining the overall healing outcome, as excessive or prolonged accumulation of inflammatory cells at this stage can delay tissue repair.^60^ In this context, the observed findings suggest that berberine may contribute to the regulation of inflammatory responses, potentially limiting excessive inflammatory cell infiltration and promoting a more favorable healing environment. This controlled modulation of inflammation may facilitate a more efficient transition to the proliferative phase and thereby improve overall wound healing.^61^^,^^62^

In the literature, locally applied BBR in rat cutaneous wound models has been reported to accelerate wound healing by enhancing physiological angiogenesis, fibroblast proliferation, and subsequent collagen production 63, 64, 65. In contrast, our study did not demonstrate statistically significant differences between the experimental and control groups in terms of fibroblast counts or vascularization. One possible explanation for this discrepancy is the pharmacokinetic limitations of BBR. Thomas et al.,^66^ reported that BBR has low bioavailability, is poorly absorbed from the gastrointestinal tract, and is rapidly eliminated. Accordingly, the dose and route of administration used in our study may have been insufficient to achieve sustained therapeutic tissue concentrations.

Thus, the discrepancy between the favorable histological outcomes reported in studies using local BBR application and our findings may be attributed to differences in administration routes and bioavailability. This indicates that further studies are needed to more clearly elucidate the effects of BBR on wound healing, particularly those using local and controlled-release formulations and evaluating different dosages.

Literature reports that berberine exhibits effects that reduce mucosal inflammation and ulceration in both systemic and local applications. In a study conducted on mice, it was found that BBR (50 mg/kg) improved intestinal mucosal damage and ulceration after oral gavage administration in an irinotecan (CPT-11)-induced intestinal mucositis model.^67^ Similarly, the fact that local application of berberine gelatin significantly reduces ulcer diameter in patients with recurrent aphthous ulcers suggests that the therapeutic efficacy of the drug is enhanced, particularly due to high local tissue concentration and prolonged contact time.^68^ In the present study, consistent with results reported in the literature, the significantly lower level of ulceration development in the experimental group on days 3 and 7 compared to the control group reveals that berberine has a significant biological effect on the onset of the early inflammatory and proliferative phases. In contrast, the decrease in the difference between the groups in later healing periods suggests that the effect of berberine may be limited to early wound healing or that sufficient and sustainable tissue levels may not have been achieved due to systemic administration. In this context, the more pronounced clinical results reported in studies using local application forms (gel, sponge, or controlled-release systems) support the idea that the bioavailability and pharmacokinetic limitations of berberine directly affect the treatment response.^69^

A study on the survival of skin flaps reported that BBR significantly reduced flap necrosis by promoting angiogenesis, suppressing inflammation, and reducing oxidative stress.^70^ In the present study, when evaluated in terms of necrosis developing in the wound area, the experimental group showed significantly lower necrosis values on day 3 compared to the control group (p = 0.058), close to the limit of statistical significance. Although this difference did not reach the level of statistical significance, this early observed trend indicates the potential effect of BBR in suppressing necrosis development. While no statistically significant difference was found between the groups in the later healing periods (days 7 and 14), considering the qualitative evaluation, the fact that three rats in the experimental groups showed no necrosis at both time points, while no rats in the control groups showed complete necrosis, suggests that BBR may have a clinically significant protective effect in the wound healing process. When these findings are evaluated holistically, the results obtained from our study support the possible positive effects of BBR on necrosis development.

When examining studies investigating the effect of BBR on edema, it is seen that there are studies conducted in different experimental models, mainly brain, paw and lung tissues 71, 72, 73. In these studies, the level of edema was mostly evaluated with measurements based on tissue weight, and it was reported that BBR statistically significantly reduced the level of edema.

In the present study, edema levels were examined using histopathological evaluation, unlike the weight-based methods commonly used in the literature. Only on day 7 of the study days was a significantly lower edema level detected in the experimental group compared to the control group. The vast majority of oral surgical procedures result in postoperative edema, and this morbidity is known to reach its maximum level in the first few days following surgery. The relatively high edema levels detected on day 7 in our study, while seemingly contradictory to clinical observations, may be related to the fact that histopathological examination can detect persistent interstitial fluid accumulation at the microscopic level and reflects a different time window than macroscopic clinical evaluations. Furthermore, the low absorption of BBR after systemic administration may have prevented sufficient efficacy in the initial days.

Therefore, while the findings of our study support the potential reducing effect of BBR on edema, they also demonstrate that different assessment methods (histopathological, weight-based, and clinical measurements) may reflect the time-varying dynamics of edema in different ways.

Our study has some limitations. Firstly, the systemic administration of BBR may have led to insufficient and unsustainable therapeutic concentrations at the tissue level compared to local application studies reported in the literature. Indeed, BBR is known to have low bioavailability after systemic administration, limited absorption from the gastrointestinal tract, and rapid elimination from the body. These pharmacokinetic properties of BBR may have prevented significant changes in serum cytokine levels.

Secondly, the use of ELISA results from serum samples to assess the inflammatory response may not have fully revealed the anti-inflammatory effects of BBR, which occur primarily at the local tissue level. Given that the anti-inflammatory effects of BBR are frequently demonstrated through tissue expression and cellular signaling pathways in the literature, the absence of tissue ELISA, immunohistochemistry, or molecular assays (such as mRNA expression) is a significant limitation of this study.

Another limitation of this study is that the histopathological evaluation was performed by a single, blinded pathologist. While this approach ensures consistency in scoring, the absence of inter-observer and intra-observer agreement analyses may limit the reproducibility of the findings. Future studies involving multiple observers and repeated evaluations are recommended.

Finally, histological evaluation of edema has made it difficult to obtain results directly comparable to clinical and weight-based measurements. The ability of histological examination to detect persistent interstitial fluid accumulation at the microscopic level may have led to different results in terms of timing compared to clinical observations.

Despite these limitations, our study provides important data on the potential positive effects of BBR on wound healing, particularly in the early inflammatory phase, and the findings are considered to be guiding for further studies using different doses, routes of administration, and local formulations.

## 5 Conclusion

This experimental study demonstrates that berberine may exert positive effects in the early stages of wound healing by limiting excessive inflammatory cell infiltration and reducing ulceration and necrosis in the early inflammatory phase. While systemic administration did not lead to statistically significant changes in serum cytokine levels, histological findings indicate that the effects of BBR are more pronounced at the local tissue level. The lack of significant differences in fibroblast proliferation and angiogenesis may be related to the pharmacokinetic limitations of systemic berberine administration. Overall, these findings highlight the potential of BBR as an adjunctive agent in wound healing, particularly during the early inflammatory phase. Future studies should focus on alternative routes of administration—such as the direct topical application of BBR to oral mucosal wounds—optimized dosing regimens, and molecular analyses at the tissue level.

## Authors’ contributions

US: Conceptualization, methodology, investigation, surgical procedures, data curation, formal analysis, writing – original draft preparation.

BE: Methodology, investigation, experimental procedures, data collection, writing – review and editing.

OC: Histopathological evaluation, microscopic analysis, data interpretation, validation.

ME: Biochemical analysis, laboratory procedures, data collection, data curation.

MK: Supervision, project administration, funding acquisition, study design, writing – review and editing.

All authors have read and approved the final version of the manuscript.

## Ethical approval

This study was approved by the Adıyaman University Local Ethics Committee for Animal Experiments (Protocol No: 2021/037; Meeting Date: 30 September 2021). All experimental procedures were conducted in accordance with the ARRIVE 2.0 guidelines and complied with institutional and national regulations for the care and use of laboratory animals.

## Clinical trial number

Not applicable.

## Consent for publication

Not applicable.

## Human and animal rights statement

This experimental animal study was conducted in accordance with the ARRIVE guidelines and the National Institutes of Health Guide for the Care and Use of Laboratory Animals.

The study protocol was approved by the Adıyaman University Animal Experiments Local Ethics Committee (Protocol No: 2021/037; Meeting Date: 30 September 2021).

All procedures were performed in compliance with institutional and national guidelines for the care and use of laboratory animals. All efforts were made to minimize animal suffering and to reduce the number of animals used in the study.

This study did not involve human participants.

## Funding

This study was supported by the Scientific Research Projects Unit of Adıyaman University (ADYU-BAP) under project number DHFDUP/2021-0005.

## Declaration of competing interest

The authors declare that they have no known competing financial interests or personal relationships that could have appeared to influence the work reported in this paper.
